# Evaluation of Psychological Distress, Self-Care, and Medication Adherence in Association with Hypertension Control

**DOI:** 10.1155/2022/7802792

**Published:** 2022-08-25

**Authors:** Maryam Eghbali, Maedeh Akbari, Kimiya Seify, Mohammad Fakhrolmobasheri, Maryam Heidarpour, Hamidreza Roohafza, Maryam Afzali, Fateme-sadat Mostafavi-esfahani, Parisa Karimian, Anis Sepehr, Davood Shafie, Alireza Khosravi

**Affiliations:** ^1^School of Nursing and Midwifery, Isfahan University of Medical Sciences, Isfahan, Iran; ^2^Hypertension Research Center, Cardiovascular Research Institute, Isfahan University of Medical Sciences, Isfahan, Iran; ^3^Heart Failure Research Center, Isfahan Cardiovascular Research Institute, Isfahan University of Medical Sciences, Isfahan, Iran; ^4^Isfahan Endocrine and Metabolism Research Center, Isfahan University of Medical Sciences, Isfahan, Iran; ^5^Cardiovascular Research Institute, Isfahan University of Medical Sciences, Isfahan, Iran; ^6^Cardiac Rehabilitation Research Center, Cardiovascular Research Institute, Isfahan University of Medical Sciences, Isfahan, Iran

## Abstract

**Background:**

Most of the patients with hypertension (HTN) who undergo medical therapy unaccompanied by psychological and behavioral interventions may not achieve their goal in HTN treatment. Self-care is a key factor in controlling HTN. Given that depression, stress, and anxiety are the most psychological disorders in chronic illnesses. Their impact on self-care, quality of life, and HTN control must be studied more.

**Methods:**

We analyzed the difference in medication adherence in 252 patients with low vs. high psychological distress. Also, patients with controlled and uncontrolled HTN were compared according to their psychological distress scores. We further assessed the relation of psychological distress, self-care, and medication adherence with patients' demographic characteristics.

**Results:**

61.3% of our participants were female with a mean age of 60.6 ± 11.35 and male participants had a mean age of 60.5 ± 11.55. The psychological distress score was significantly higher in women with uncontrolled HTN (*p* value = 0.044). Also, individuals with controlled HTN tend to have a higher medication adherence score (*p* value = 0.01) and higher self-care score (*p* value = 0.033). Hypertensive females had a higher psychological distress score (3.35 ± 2.05) and a lower self-care score (64.05 ± 8.16). There was a positive relationship between age and drug adherence. The self-care score was higher (65.95 ± 7.88) in patients having lower psychological distress levels.

**Conclusion:**

A lower psychological distress score can result in better self-care, enhancing the probability of better HTN control; thus, psychological interventions may be necessary for the treatment of HTN. However, more studies are needed to assess the effectiveness of this intervention.

## 1. Introduction

Hypertension (HTN) is one of the greatest healthcare concerns worldwide [1]. As a multifactorial disease, HTN is affected by both genetic and environmental factors such as age, sex, educational status, physical activity, smoking, body mass index (BMI), and history of diabetes mellitus (DM) [2]. It is estimated that one-third of adults are hypertensive worldwide. According to predictions, one billion individuals will be hypertensive by 2025 [3]. HTN is the third cause of disability in the elderly population. It is a major contributor to the development of coronary artery disease (CAD), heart failure (HF), cerebrovascular accident (CVA), and chronic kidney disease (CKD). HTN is the cause of 9.4 million deaths per year and also the cause of 45% of chronic heart diseases and 51% of CVAs leading to death [1, 3].

HTN is a chronic disease requiring long-term strategies to be managed appropriately. Previous studies indicated that approximately 70% of hypertensive patients would not reach a favorable blood pressure (BP) control if they receive medical treatment as the only treatment strategy [4–6]. Multiple factors could affect medical and nonmedical adherence, such as age, race, social support, anxiety, and depression [7]. Proper management for HTN is reachable within the accompany of patients and health care providers [8]. Self-care is defined as the capabilities of patients and communities to involve in the activities related to health promotion and disease prevention and management.

Regarding the role of self-care in the management of HTN, medication adherence, physical activities, healthy diet, weight control, stress management, reducing alcohol use, and tobacco avoidance are behaviors known to be associated with better BP control [9]. Studies demonstrated that good self-care could result in up to 5 mmHg reduction in systolic and 4.5 mmHg reduction in diastolic BP. Although self-care has an established role in HTN control and is of the essential steps for managing HTN, only a few individuals may follow these lifestyle recommendations [10]. The Eighth Joint National Committee (JNC-8) has eight suggestions for HTN self-care, one of which is anxiety and stress management [11]. Among all factors and behaviors associated with self-care in HTN patients, psychological factors may be of greater importance. Psychological distress could be the cause or the consequence of a chronic cardiovascular disorder [7, 12]. Numerous studies indicated the relation between psychological distresses with HTN [13]. Moreover, it has been reported that individuals suffering psychological distress and HTN simultaneously are at a higher risk for cardiovascular mortality compared to hypertensive patients without psychological distress [14].

Long-term psychological intervention could improve the patient's quality of life and help control HTN better. It also reduces the risk of CVAs in hypertensive patients [15]. Depression, stress, and anxiety are the most common psychological disorders in chronic illnesses. Assessing the impact of such conditions on the patients' self-care, quality of life, and disease control could arm the healthcare systems with more information, developing more precise and efficient multifactorial treatment strategies [14]. Here, in this study, we evaluated psychological distress in hypertensive patients. Furthermore, we evaluated the differences in self-care scores in patients with low and high psychological distress and patients with controlled and uncontrolled HTN. We also compared the psychological distress score in controlled and uncontrolled HTN.

## 2. Methods

### 2.1. Study Design and Protocol

“Trends of prevalence, awareness, treatment, and control hypertension and the effect of expanded chronic care model on control, treatment, and self-care” is a multistage program performed on the general population of Isfahan, Iran, which was conducted by the Isfahan Cardiovascular Research Institute (a regional World Health Organization collaborative center) [16]. During the first phase of the program, in a 10-month period, a validated questionnaire was developed attempting to obtain information regarding participants' demographics, knowledge, attitude, practice, self-care, psychological status, and medication adherence. During the second phase, in 7 months, 1818 subjects were included in the study using the multistage random cluster sampling method. The study population was recruited in order to reflect the demographic and socioeconomic status of the targeted population (general population of Isfahan, Iran). During the procedure of the 2^nd^ study phase, after obtaining written informed consent, measurements and the interview process (using the predefined questionnaire in the first phase) were performed. The third phase included two groups nonrandomized clinical trial with before and after design (intervention and control groups): the aim of this stage was to assess the effect of the Expanded Chronic Care Model (ECCM) on HTN control, management, and patients' self-care. The final phase of the study consisted of a comparative study on the prevalence and risk factors associated with HTN in Isfahan between the mentioned 4 phase study and the prior studies in this field. The current study is an analysis of the results from the second phase of the “Trends of prevalence, awareness, treatment, and control hypertension and the effect of expanded chronic care model on control, treatment, and self-care” study.

### 2.2. Sample Size Selection

In this study, the prevalence of HTN in the total population was considered 18.9% [17]. We assumed type 1 error as *α* = 0.05 and the margin of error as *d* = 0.018. Using the Equation (1), we calculated the sample size as 1818 persons considering *Z* value = 1.96. Furthermore, sampling was performed using a multistage clustered sampling method from the 18 health centers of Isfahan, Iran. The samples were selected according to the demographic structure of the target population including age, sex, and socioeconomic status of the target population.

(1) $$\begin{array}{c} n=\frac{Z^{2}P\left(1-P\right)}{d^{2}}, \end{array}$$

Equation (1) is the sample size calculation formula.

### 2.3. Inclusion and Exclusion Criteria

Every individual aged 18 or more living in Isfahan with a history of HTN is deemed fit for this study. The exclusion criteria included casting, shunts, fistula, or any other limitations for measuring BP, fasting or any special diets, pregnancy, dialysis, Cushing syndrome, pheochromocytoma, cancer, and presence of mental illnesses. The detailed study protocol and sampling method are published separately [16].

### 2.4. Study Procedure and Measurements

The study's protocol was explained to the participants on arrival at the health care center and written informed consent was obtained. Then, while being site, they were asked about their demographic and socioeconomic information, including age, sex, years of education, marital status, and occupation status. Also, a brief history regarding DM, dyslipidemia, smoking, family history, and years was taken since they were diagnosed with HTN. Afterward, participants' medication adherence, psychological distress, and self-care were assessed. Participants then rested in a quiet room for five minutes, and then, their BP was measured three times at one-minute intervals from each arm. The BP was measured using WHO standard measures. We defined uncontrolled HTN as blood pressure higher than 140/90 with or without pharmacological treatment. The mean of second and third measured BP was considered patients' BP in this study. The BP was measured by a digital arm BP tool calibrated with a standard mercury sphygmomanometer several times on 1 to 3 persons. After that, individuals' height and weight were collected and their BMI was calculated.

### 2.5. Questionnaires

We used the 8-item Morisky Medication Adherence Scale (MMAS-8) to assess medication adherence. This questionnaire contains eight questions, seven of which have two choices (yes/no) and one is a Likert-type question with an answer selection of never, rarely, sometimes, usually, and all the time. Each question has one point. Participants were categorized based on points to low (<6), moderate [6, 7], and high [8] adherence groups. The validity and reliability of MMAS-8 were tested and verified in a previous study [18]. Psychological distress was evaluated with the General Health Questionnaire (GHQ-12). This is a self-reporting 12 item questionnaire with a four-point scaling system (less than usual, no more than usual, fairly more than usual, or much more than usual). The 0-0-1-1 method was used to score the GHQ-12 questions in this study. Less than usual and no more than usual got zero points and fairly more than usual or much more than usual got one point. Participants with a score of 3 or less were classified as without psychological distress. A score of four to six was classified as low psychological stress, and a score of seven or more indicated a high psychological stress patient. We also used a cutoff value of ≥4 to indicate psychological distress in an individual. The validity and reliability of this questionnaire were tested and verified in previous studies [19–21]. To assess self-care, we used a combination of the WHO STEP wise approach to chronic disease risk factor surveillance (STEPS) questionnaire and national questionnaires. Eventually, we designed a 16-item questionnaire with Cronbach's alpha coefficient of 0.833 that evaluated follow-up, lifestyle, support system, medication, and avoiding environmental risk factors [16].

### 2.6. Statistical Analysis

The collected data were saved in Epi info software after sending the questionnaires to the Isfahan hypertension research center. The analysis was done using Statistical Package for the Social Sciences (SPSS) version 28. In order to compare the means between groups, we used the independent sample *T* test and paired sample *T* test. We also used Pearson's correlation test to evaluate the linear correlation between numerical variables. A *p* value below 0.05 was considered significant.

## 3. Results

Of the 1818 included participants in the second phase of the study, 252 individuals were hypertensive. Eventually, data from 252 individuals were collected in this study. 61.3% of participants were female with a mean age of 60.6 ± 11.35 and male participants had a mean age of 60.5 ± 11.55. BMI had an average of 30.44 ± 4.52 in women, significantly higher (*p* value = 0.003) than in men (28.61 ± 3.62). Women mainly were housekeepers, smoked less, and had a lower marital rate than men. They had been diagnosed with HTN longer and suffered more from dyslipidemia than men (Table 1).

As demonstrated in Table 2, the psychological distress score was significantly higher in women with uncontrolled HTN (*p* value = 0.044) (Table 2). Also, individuals with controlled HTN tend to have higher medication adherence scores (*p* value = 0.010) and higher self-care scores (*p* value = 0.033) (Table 3).

Table 4 demonstrates the association of drug adherence, psychological distress, and self-care with age, sex, years of education, and daily physical activity.

Regarding drug adherence, it had a weak but positive correlation with age (R = 0.217, *p* value = 0.001). Patients with regular daily physical activity were significantly more adherent to the drugs (5.74 ± 2.53 vs 4.86 ± 2.69, *p* value < 0.001); nevertheless, drug adherence was not associated with sex and years of education.

Our analysis of psychological distress indicated that female participants had marginally higher psychological distress (3.35 ± 2.05 vs 2.82 ± 2.20, *p* value = 0.049) and psychological distress correlated negatively with years of education (R = -0.140, *p* value = 0.026); however, this was not associated with age and daily physical activity.

Regarding self-care, men had a significantly higher self-care score (66.50 ± 7.87 vs 64.05 ± 8.16 *p* value = 0.017); also, participants with regular daily physical activity had higher self-care scores (67.05 ± 7.15 vs 62.83 ± 8.62, *p* value = 0.008) but there was no significant association between self-care, age, and years of education (Table 4).

Table 5 shows the relation between psychological distress and self-care score. The mean self-care scores were higher (65.95 ± 7.88) in patients having lower psychological distress levels (Table 5).

## 4. Discussion

The growing prevalence of HTN, particularly after the recent pandemic, and the growing economic burden of the disease highlight the importance of studies surrounding the contributing factors and management strategies of chronic diseases [3, 22]. Nonmedical interventions such as self-care development and stress management are the major parts of the long-term plan for HTN management [4]. Thus, it is essential to evaluate psychological distress as a determining factor affecting self-care, medication adherence, and HTN control.

Hypertension, as one of the greatest healthcare concerns worldwide, requires a multidisciplinary approach involving physicians, dietitians, nurses, psychotherapists, and pharmacists [23]. Prior studies have proposed psychological distress as a risk factor for the development of hypertension and it has been shown that hypertensive patients may struggle with more psychological distress in life [12]. Psychological distress could affect the patient's self-care resulting in lower medication adherence and an unhealthy lifestyle [24]. Studies on patients with cardiovascular disorders, particularly heart failure, indicated that patients may not follow the principles of self-care unless their psychological distress improved by proper psychotherapeutic interventions [25].

Our study indicates that the prevalence of psychological distress, based on the GHQ-12 questionnaire, was 34.1% in our study population which is higher than the reported prevalence from previous studies in Nigeria and England (10.8 and 15.7%, respectively) [26, 27] but was close to a report from hypertensive patients in Malaysia(28.8%) [28]. The higher rate of psychological distress among our study population might be due to the demographic differences and the increasing challenges related to globalization and socioeconomic alterations in the studied region [29]. Moreover, in line with previous studies, our findings demonstrated that patients with higher self-care scores had significantly lower psychological distress levels; however, previous studies associating self-care with psychological distress were mostly about other conditions including diabetes and ischemic heart diseases [30, 31].

Moreover, our findings introduced educational status as another factor significantly associated with psychological distress which was consistent with previous studies in this field [32, 33]. The relationship between psychological distress and educational levels might be explained by the fact that the more educated individuals may have greater insights into their psychological condition and have a more positive view of mental health thus, might seek help to control their psychological distresses [33].

Our results also represented that psychological distresses were more prevalent in women which could be justified because women in our study population had lower education compared to men and were primarily housewives; however, this finding may vary considerably in different regions [34]. We also found that the female population in our study was more likely to be single, divorced, or widowed which could also explain the higher prevalence of psychological distress in females [35]. Nevertheless, other studies also indicated that the female population is more prone to experience psychological distress. Matud et al. in a study in Spain proposed that spending more time on housekeeping and less time to do enjoyable activities may cause higher psychological distress perceived by women [36]. In another study by Viertiö et al., it was demonstrated that feelings of inadequacy as a parent are greater in women when they are absorbed in their work [37].

In line with our result, Chen et al. in a study on 220 hypertensive individuals postulated that psychological wellbeing plays a mediating role between knowledge of self-care and self-care behavior [38]. Furthermore, studies also indicated that psychological wellbeing is a mediator of the effects of social and family support in patients with hypertension [39]. Moreover, in a systematic review, it has been shown that managing depression could effectively lower blood pressure in hypertensive patients with depression [40].

Regarding medication adherence, our study revealed two factors (age and physical activity) affecting medication adherence. Interestingly, we found that age has a positive correlation with medication adherence. This finding supports the idea that patients aging 65 to 80 may have greater medication adherence compared to younger or older individuals [41]. This idea may be supported by the fact that younger individuals may spend more time at work and experience more daily stress and thus have poor adherence to medical treatments [42]. Physical activity, as the second factor associated with medication adherence, is closely related to psychological wellbeing. It has been shown that regular physical activity could improve the psychological status and indirectly affect medication adherence [43]. However, in the current study we could not indicate the direct association between physical activity and psychological distress.

Taken together, our results support the findings on the role of psychological distress as a mediator for self-care and medication adherence in hypertensive patients. Patients with better psychological status are more likely to adhere to medical treatment and have better self-care thus; they may have better controlled blood pressure. We also indicate that female individuals and those with the lower educational backgrounds are more likely to suffer from psychological distress. Accordingly, these subgroups of hypertensive patients may need more consideration regarding their mental health.

This study was not free from limitations. The cross-sectional design prevented us from determining direct casualty relations. Accurate BP, weight, and height measurements were of the points strength. Moreover, during the sampling procedures, which are described comprehensively separately [16], we attempted to also include households that did not have any pre-existing documents in their nearby healthcare centres. This work was done in order to decrease bias.

## 5. Conclusion

In conclusion, lower psychological distress can result in better self-care, enhancing the probability of optimal HTN control. Regarding the importance of HTN and considering the increase of stressors, studying factors improving patients' psychological status can benefit public health.

## Figures and Tables

**Table 1:** The demographic and medical characteristics of the study population divided by sex. A *p* value < 0.05 was considered significant.

	*Mean (standard deviation)*			*p* value
	Female	Male		
Age (years)	60.60 ± 11.35	60.50 ± 11.55		0.854
Years of education	4.97 ± 4.91	8.73 ± 5.41		0.109
BMI	30.44 ± 4.52	28.61 ± 3.62		0.003
Years since hypertension diagnosed	7.67 ± 6.87	5.35 ± 4.27		<0.001
SBP	134.38 ± 19.78	136.71 ± 16.26		0.031
DBP	77.88 ± 11.92	81.47 ± 10.74		0.204
MAP	96.71 ± 13.18	99.88 ± 11.31		0.040

*Numbers (%)*				*p* value
Sex	252 (100%)	138 (61.3%)	114 (38.7%)	
*Marital status*	Married	97 (70.3%)	107 (93.9%)	<0.001
	Single/divorced/dead	41 (29.7%)	7 (6.1%)	
*Occupation*	Employed	4 (2.9%)	45 (39.5%)	<0.001
	Housekeeper	128 (92.8%)	0 (0.0%)	
	Retired	6 (4.3%)	68 (59.6%)	
	Student/unemployed	0 (0.0%)	1 (0.9%)	
*Diabetes*	Yes	51 (37%)	35 (30.7%)	0.182
	No	87 (63%)	79 (69.3%)	
*Hyperlipidemia*	Yes	78 (56.5%)	50 (43.9%)	0.030
	No	60 (43.5%)	64 (56.1%)	
*Smoking*	Yes	3 (2.2%)	22 (19.3%)	<0.001
	No	135 (97.8%)	92 (80.7%)	
*Family history*	Yes	90 (65.2%)	67 (58.8%)	0.179
	No	48 (34.8%)	47 (41.2%)	

**Table 2:** The mean of psychological distress score in people with controlled and uncontrolled hypertension. A *p* value < 0.05 was considered significant.

Hypertension		Controlled	Uncontrolled	*p* value
*Mean of psychological distress score*	All	3.05 ± 2.07	3.19 ± 2.22	0.615
	Women	3.08 ± 1.72	3.80 ± 2.45	0.044
	Men	3.03 ± 2.48	2.55 ± 1.74	0.252

**Table 3:** The mean of drug adherence and self-care score in the individual with controlled and uncontrolled hypertension. A *p* value < 0.05 was considered significant.

Hypertension	Controlled	Uncontrolled	*p* value
Mean of drug adherence score	5.70 ± 2.45	4.83 ± 2.82	0.010
Mean of self-care score	66.04 ± 7.51	63.83 ± 8.80	0.033

**Table 4:** Comparison of the scores of drug adherence, psychological distress, and self-care with age, sex, years of education, and regular daily physical activity. A *p* value < 0.05 was considered significant.

Personal and clinical characteristics		Drug adherence	*p* value	*R*	Psychological distress	*p* value	*R*	Self-care	*p* value	*R*
*Sex*	Female	55.55 ± 2.57	0.192	—	3.35 ± 2.05	**0.049**	—	64.05 ± 8.16	**0.017**	—
	Male	5.11 ± 2.70			2.82 ± 2.20			66.50 ± 7.87		
Age (years)		—	**≤0.01**	+0.217	—	0.651	−0.029	—	0.335	+0.061
Years of education		—	0.193	-0.082	—	**0.026**	−0.140	—	0.122	+0.098
*Physical activity (3–5 times/week)*	Yes	5.74 ± 2.53	**≤0.01**	—	3.11 ± 2.07	1.00	—	67.05 ± 7.15	**0.008**	—
	No	4.86 ± 2.69			3.11 ± 2.21			62.83 ± 8.62		

**Table 5:** The relation between the level of psychological distress and self-care score. A *p* value < 0.05 was considered significant.

Level of psychological distress	Self-care score (mean and standard deviation)	*p* value
Low (*n* = 166)	65.95 ± 7.88	**0.031**
High (*n* = 86)	63.62 ± 8.36	

## Data Availability

All data used to support the findings of this study are available on reasonable request to the corresponding author.
